# What is Collaborative Care? Reexamining the Principles of Collaborative Care Through Scaling to Complex Populations and Settings

**DOI:** 10.1007/s11606-026-10552-x

**Published:** 2026-06-15

**Authors:** Grace M. Hindmarch, Alex R. Dopp, Sapna J. Mendon-Plasek, Katherine E. Watkins

**Affiliations:** 1 RAND, 1776 Main Street, Santa Monica, CA 90407-2138 USA; 2 University of California Los Angeles, Fielding School of Public Health, Department of Health Policy and Management, 650 Charles E Young Dr S, Los Angeles, CA 90095 USA

## Abstract

The Collaborative Care Model (CoCM) is a principle-based, behavioral health integration model used in primary care to improve access and quality of behavioral health treatment. CoCM was originally developed and tested for the treatment of depression. This JGIM Perspective argues that when principle-based models are scaled to complex populations and settings beyond those for which they were originally developed, the boundaries of the principles are strained. In scaling CoCM for the treatment of opioid use disorder (OUD) co-occurring with PTSD and/or depression in a randomized trial, we found challenges with enacting the principles of CoCM for a complex population in low-resourced settings. Key considerations included understanding the scope of the care manager role while adapting practices for low-resourced settings (e.g., using community health workers (CHWs) in the role; delivery of psychotherapy by CHWs), understanding if and how to address patient social needs in CoCM care plans, overcoming lack of validated measures for OUD symptoms, and understanding the appropriate timeframe and supports needed to provide population-based care to complex patients with co-occurring disorders. Practitioners, researchers, and policymakers should collaborate to refine understanding of the CoCM principles to strengthen the foundation of CoCM and its application to various populations and settings.

*Imagine you have been charged with implementing the Collaborative Care Model (CoCM) to improve access to behavioral health care. Your organization wants to implement CoCM broadly to reach all patients who might benefit, while still considering resource limitations. You review the literature, trying to learn how to develop a program for patients with varied clinical and social needs. You realize there are many different descriptions of CoCM—how can you be sure you’re effectively putting its principles into action?*

CoCM is a principle-based behavioral health integration model, widely researched and implemented for its ability to improve behavioral health treatment access and quality in primary care.^1^ The model consists of a multi-professional team delivering measurement and evidence-based care to a population of patients with common behavioral health disorders. The model adds a care manager and a psychiatric consultant to the care team; the care manager assesses patient symptoms using standardized measures, receives clinical consultation from a psychiatric consultant, and coordinates treatment. In some models, the care manager provides psychotherapy.

Principle-based interventions are useful for addressing complex health conditions but are difficult to define precisely. In such interventions, overarching principles outline the core functions needed for intervention effectiveness while allowing maximum flexibility in the forms (e.g., actions, tools) that intervention delivery takes for specific patients and settings.^2^ The original foundation for CoCM’s principles was chronic disease management models, including the Chronic Care Model,^3–5^ that were applied to improve the treatment of depression in primary care.^6^ Since then, CoCM has been applied to anxiety disorders, posttraumatic stress disorder (PTSD), bipolar disorder, and substance use disorders, resulting in a variety of practices, all called collaborative care.^7–10^ Table 1 summarizes the CoCM principles.

**Table 1:** Principles of the Collaborative Care Model

(1)	A multi-professional team approach to patient care with clearly defined roles, including a care manager who acts as the link between the primary care provider, the behavioral health provider, and the psychiatric consultant, and who helps the team deliver population-based, evidence-driven behavioral health care
(2)	A structured patient care management plan that includes provision of evidence-based interventions
(3)	An organized approach to proactive patient follow-ups that (a) encourages adherence to the management plan and (b) monitors response to interventions using validated measures
(4)	Enhanced inter-professional communication that facilitates population-based care by teams, including tools (e.g., a patient registry) and systems (e.g., access to specialist expertise via consultation)

*Source*: Systematic reviews of the Collaborative Care Model,^5,7^ with language incorporated to reflect the original Chronic Care Model^3,4^ and a more recent expert consensus on Collaborative Care^1^

In our experience, scaling out CoCM beyond the populations and settings for which it was originally developed tested its core principles^11^ by revealing (and causing us to reexamine) assumptions underlying those principles. Regarding population, we applied CoCM to a complex patient population for which CoCM was not initially designed: individuals with co-occurring opioid use disorder and mental illness, high social needs, and chronic pain. Reconciling the multiple, competing needs of these patients with CoCM’s intensive focus on a single condition was challenging. Regarding setting, we delivered CoCM in clinics serving primarily low-income patients and located in provider shortage areas, which challenged us to reconsider the minimum qualifications and supports needed for the care team (i.e., we used community health workers (CHWs) in the role of the care manager). It is rare to see reflection on how specific applications of CoCM relate back to its foundational principles; for this Perspective, we engaged in such reflection to refine our understanding of enacting CoCM principles when scaling out to different populations and/or settings.

## CASE EXAMPLE: THE CLARO CLINICAL TRIAL

**Figure 1:**
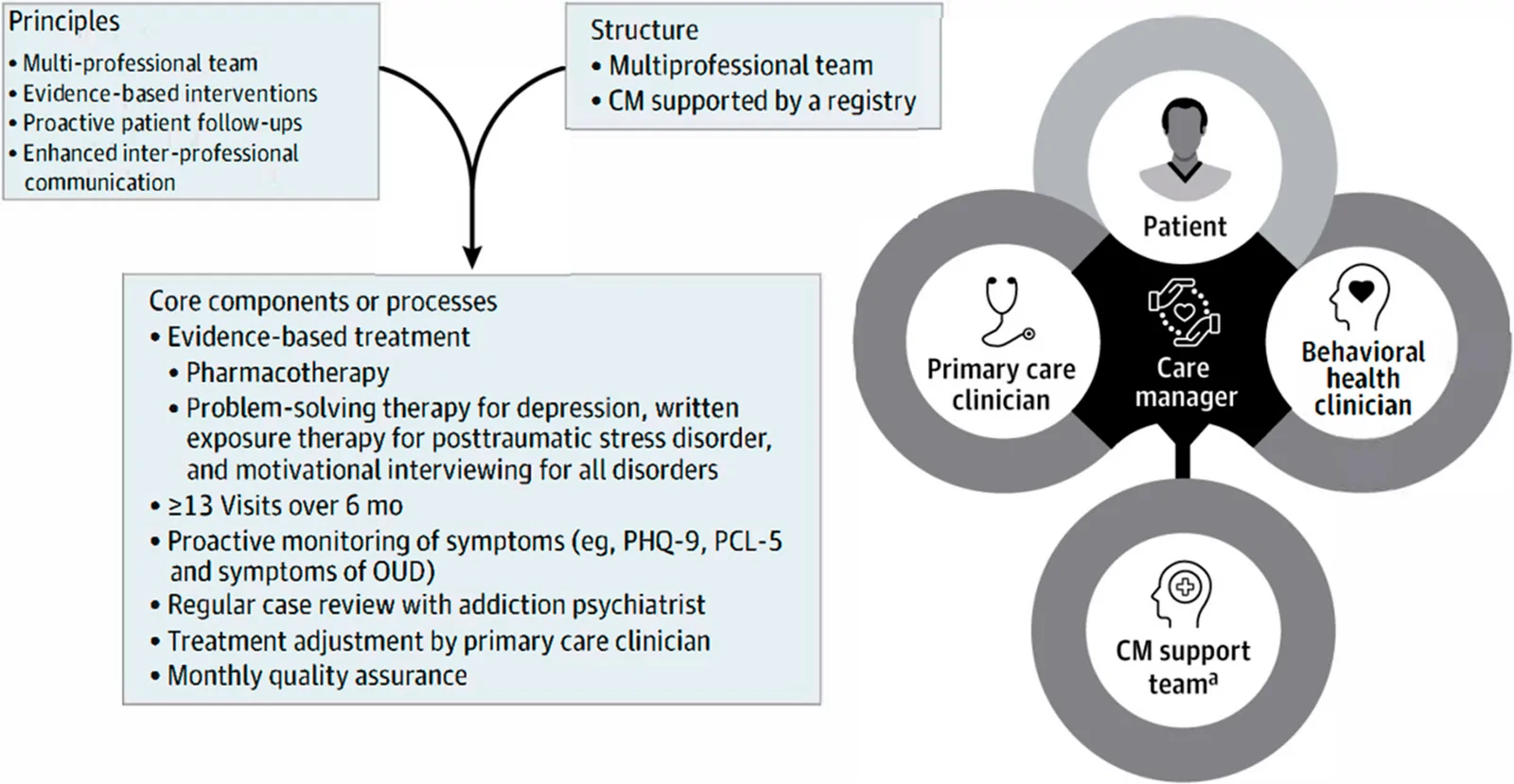
The CLARO Collaborative Care Model. *Source*: The Figure is adapted from Watkins KE, Osilla KC, McCullough CM, Griffin BA, Dopp AR, Becker K, et al. Collaborative Care for Opioid Use Disorder and Mental Illness: The CLARO Randomized Clinical Trial. JAMA Internal Medicine. 2026;186(2):168–80. CM indicates care manager; PCL-5, Post-Traumatic Stress Disorder Checklist for DSM-5; PHQ-9, Patient Health Questionnaire-9. ^a^Support team includes an addiction psychiatrist, a supervising psychologist, and a supervising community health worker.

CLARO (Collaboration Leading to Addiction treatment and Recovery from Other stresses) tested CoCM for the treatment of OUD co-occurring with PTSD and/or depression in low-resourced primary care settings.^12–14^ Figure 1 summarizes how CLARO operationalized the principles, structure, and core components of CoCM^15^ and Table 2 details how CLARO operationalized each principle. We used CLARO as a case example to reflect on challenges enacting CoCM principles with complex populations and settings and had informal conversations with CoCM experts in our network (see Acknowledgements) about such challenges; these conversations informed this Perspective but were not formal qualitative interviews.

**Table 2:** How CoCM Principles were Operationalized for CLARO

(1)	Multi-professional team approach to patient care
	• Community health workers performing care manager role
	• Primary care providers overseeing care and providing medication treatments
	• Behavioral health providers delivering psychosocial treatments
(2)	Care management plan with evidence-based interventions
	• Medication treatment for OUD
	• Problem-Solving Therapy and/or medication for depression
	• Written Exposure Therapy and/or medication for PTSD
	• Screening patients for social needs, and referring them to local resources as needed
(3)	Proactive patient follow-ups
	• Care manager initiation visit and up to 12 follow-up visits over 6-month period
	• Use of newly adapted, standardized measure to track OUD symptoms
	• Use of validated measures to track depression and/or PTSD symptoms
	• Care manager reaches out to engage patients who miss scheduled appointments
(4)	Enhanced inter-professional communication for population-based care
	• Patient registry that tracks progress with OUD, depression, and PTSD symptoms and treatments, as well as social needs
	• Caseload review with psychiatric consultant who has addiction medicine expertise; supplemented by care manager support team with community health worker expertise

*Source*: Summarized from publications documenting the CLARO clinical trial^12,14,15^

*Note*: See Table 1 for details of each principle. OUD = opioid use disorder, PTSD = posttraumatic stress disorder

### Multi-professional Team Approach to Patient Care

This principle involves primary care providers and other health professionals working together on a shared care management plan. CoCM adds a care manager role that coordinates the care team, enabling task shifting to increase team efficiency and expanding the scope of care (e.g. more attention on patient adherence). Driven by input from our partner clinics (most clinics were in Health and/or Mental Health Professional Shortage Areas), we used CHWs to mitigate shortages of licensed providers who could serve as care managers, such as nurses or social workers. We also used CHWs due to their strengths in patient engagement^16^; challenges engaging patients is a known barrier for patients with OUD.^17^ Prior research supports the effectiveness of lay practitioners working under the supervision of licensed providers to deliver behavioral health care.^18,19^ Finally, our partner clinics had already established CHWs roles and funding sources, which supported sustainability.

Although the role of the care manager often includes delivering psychotherapy, in CLARO, CHWs referred patients to behavioral health providers for psychotherapy. Therapists were typically part of the primary care clinic, except for one health system where CHWs could only refer to an external community-based organization. Referring patients to other providers for psychotherapy was challenging, and many patients did not receive psychotherapy.

CoCM experts agreed that CoCM principles do not specify who provides which treatment, nor which types of professionals are in the care manager role. Several believed that a CHW would not be appropriate as a care manager because of a lack of training and credentialing to deliver evidence-based psychotherapies, and that referring patients out for psychotherapy was not consistent with CoCM principles. Others disagreed and thought that anyone could be the care manager if they had the requisite training and supervision, and could refer patients for psychotherapy.

Given the lack of consensus, it will be useful to continue exploring how care manager roles can be defined for a variety of workers (including but not limited to CHWs). Although many CoCM experts suggested that the care manager should provide psychotherapy, for communities in provider-shortage areas, this may not be possible. In current work, we are examining the feasibility, appropriateness, and effectiveness of CHWs delivering brief psychosocial interventions.

### Evidence-based Care Management Plan

Evidence-based care plans emphasize that patients receive treatments with evidence supporting their effectiveness. A recent meta-analysis found the most influential component of CoCM for reducing depression symptoms was the therapeutic treatment strategy, especially use of manual-based psychotherapy.^20^ In CLARO, evidence-based medications for OUD, PTSD, and depression were provided by primary care providers; psychotherapy, including Written Exposure Therapy for PTSD and Problem-Solving Therapy for depression, was delivered by behavioral health providers.

All CLARO patients had OUD and co-occurring depression and/or PTSD, and many had chronic pain, physical health disorders, and high social needs.^13^ Given limited time, care managers needed to prioritize what to focus on, considering the relative importance of addressing each disorder and social needs. CHWs, because of their training and familiarity with addressing social needs, may have been more likely to focus on these, which could have limited attention to OUD and mental health problems. In the context of patient complexity, how to prioritize competing issues remains challenging.

The CoCM experts disagreed about whether social needs should be addressed as part of collaborative care. Some believed that focusing on social needs would take away from measurement-based care and time to deliver psychotherapies; others felt that effective clinical care cannot be separated from addressing patients’ social needs. The experts acknowledged that the current CoCM principles do not offer guidance on when and how care management plans should address social needs, or how to prioritize between different clinical conditions.

The original Chronic Care Model included “Community resources and policies,” which emphasized that primary care clinics need linkages with community-based resources to fully meet patient needs.^3^ In CLARO, group supervision for care coordinators facilitated knowledge-sharing and troubleshooting challenges around matching patient needs with available resources. Future CoCM implementation could consider similar practices. There is room for learning how social needs fit into the model, both conceptually and from a policy perspective. It would be useful to empirically test the benefits of addressing social needs (e.g., through community linkages) in improving outcomes, and to update the principles as warranted.^21^

### Proactive Patient Follow-ups Within Population-Based Care

In population-based care, the care manager tracks a population of patients using a registry; all patients are followed even if they miss appointments. Patients’ symptoms are regularly monitored using validated measures, so that the team can adjust treatment as needed. In CLARO, care managers met with patients over 6-month periods and used a registry to proactively reach out to patients who missed appointments, tracked PTSD and depression symptoms using validated measures, tracked OUD symptoms with a newly adapted measure, and conducted caseload reviews with a psychiatric consultant. Many modifications for CLARO cut across the principles of population-based care (e.g., adding measures for all relevant diagnoses to the registry) and proactive follow-ups (e.g., tracking those symptoms regularly), so we discuss them together.

One notable limitation in CLARO was that no measurement-based care tools for OUD existed when we started the study. An important opportunity for improving CoCM is the creation of more measurement-based care tools for a wide variety of behavioral health problems, including OUD. Additionally, the principles do not provide guidance on how to prioritize measurement-based care for patients with multiple comorbidities. In CLARO we found that care managers could not assess or support care activities for all conditions at every visit, necessitating prioritization.

Several CoCM experts stated that measurement-based care is the key benefit that CoCM adds over usual primary care. They also acknowledged that delivering CoCM for complex populations is more challenging because patients have many needs to be addressed and may take longer to engage in care. Further, the experts generally found it difficult to imagine a care manager balancing patient engagement, care coordination, delivery of evidence-based care for co-occurring conditions, symptom monitoring, and addressing social needs in the short timeframe of a typical encounter. Some experts believed that a lack of improvement in symptoms in the short timeframe typical of the CoCM intervention would suggest that a patient should be referred to a higher level of care. However, length of the intervention is not specified in the CoCM principles. It will be important to explore expanded teams (e.g., multiple care managers), longer timeframes for intervention, or both when applying CoCM to complex populations. Indeed, a nurse practitioner involved in CLARO routinely advocated for providing care management to patients beyond the 6-month trial intervention period, and care managers sometimes observed notable patient progress after the formal end of the intervention.

## FUTURE DIRECTIONS

Our experience delivering CoCM for complex populations and settings challenged our understanding of the prevailing consensus on CoCM principles. Examples of key issues include the scope of the care manager role within the multi-professional team; whether the care manager should address patients’ social needs; creation of validated tools for measurement-based care; and exploration of the timeframe and supports needed to provide population-based care for complex patients. For example, CLARO employed CHWs to deliver CoCM as a strategy to address workforce shortages. However, this meant that care managers did not provide brief psychosocial interventions as is typical in CoCM. Challenges with referring patients to psychotherapy in an environment with workforce shortages highlight that more work is needed to successfully implement CoCM in low-resourced settings, potentially by expanding CHWs’ roles. Future research and policy should aim to address challenges providing behavioral healthcare in low-resourced settings, including studying the feasibility and effectiveness of CHWs providing brief psychosocial interventions.

Joint understanding of the CoCM principles with policymakers—inclusive of diverse roles such as health system administrators, funders like the U.S. Centers for Medicare and Medicaid Services (CMS),^22^ etc.—will be critical. Service structures and incentives put in place by policymakers directly influence how principle-based intervention models are implemented and sustained in practice, as has been observed for other models such as wraparound^23^ and multisystemic therapy.^24^ In the CLARO trial, we estimated that half the time spent by care managers would have been unreimbursable under CMS billing codes, a major threat to implementation and sustainability.^25^ Again, the Chronic Care Model recognized such issues by including “Health care organization” as a key principle, emphasizing that leaders need to view the populations and services as a priority and that the funding environment is highly influential.

In sum, our work illustrates why it will be useful to practitioners, researchers, and policymakers to collaborate around refined understanding of the CoCM principles. Such efforts would strengthen the foundation of CoCM and the application of its principles to various populations and settings.

## Data Availability

Data sharing is not applicable to this article as no datasets were generated or analyzed during the current study.
